# Covid-19 and child criminal exploitation in the UK: implications of the pandemic for county lines

**DOI:** 10.1007/s12117-021-09442-x

**Published:** 2021-12-06

**Authors:** Ben Brewster, Grace Robinson, Bernard W. Silverman, Dave Walsh

**Affiliations:** 1 Rights Lab, University of Nottingham, Highfield House, University Park, Nottingham, NG7 2RD UK; 2 De Montfort University School of Law, Vaughan Wy, Leicester, LE1 5XZ UK

**Keywords:** Organised crime, Drug supply, Law enforcement, Safeguarding, Covid-19

## Abstract

In March 2020, the UK was placed in lockdown following the spread of the Covid-19 virus. Just as legitimate workplaces made changes to enable their employees to work from home, the illicit drugs trade also made alternative arrangements, adapting its supply models to ensure continuity of operations. Based upon qualitative interviews with 46 practitioners, this paper assesses how front-line professionals have experienced and perceived the impact of Covid-19 on child criminal exploitation and County Lines drug supply in the UK. Throughout the paper, we highlight perceived adaptations to the County Lines supply model, the impact of lockdown restrictions on detection and law enforcement activities aimed at County Lines, and on efforts to safeguard children and young people from criminal exploitation. Our participants generally believed that the pandemic had induced shifts to County Lines that reflected an ongoing evolution of the drug supply model and changes in understanding or attention because of Covid-19 restrictions, rather than a complete reconstitution of the model itself. Practitioners perceived that Covid-19 has had, and continues to have, a significant impact on some young people’s vulnerability to exploitation, on the way in which police and frontline practitioners respond to County Lines and child criminal exploitation, and on the way illegal drugs are being moved and sold.

## Introduction

The onset of the Covid-19 pandemic in March 2020 was closely followed by substantial media speculation (Eastwood et al. 2020; Grierson and Walker 2020; Pidd 2020; Tidy 2020), as well as reports from frontline practitioners (National Youth Agency 2020; Wedlock and Melina 2020), that lockdown restrictions imposed to stem rates of infection were having a clear impact on ‘County Lines’ drug distribution and child criminal exploitation in the UK. National lockdowns and other restrictions limited people’s capacity to move freely without creating suspicion, and reports suggested that the County Lines distribution model, which typically relies on the transportation of drugs and money between larger metropolitan and provincial or coastal areas, had been disrupted during periods of national lockdown (Caluori 2020).

The perceived impact of the pandemic also extended beyond the business side of drug supply. Reductions in reports of missing children were initially taken as a sign that fewer young people were being exploited through County Lines (Caluori 2020), while other reports hypothesised that drug distribution networks may have instead developed a variety of new approaches and supply tactics to avoid detection by police (Caluori 2020; Pidd 2020; Saggers 2020a, 2020b). Youth justice, youth work and child protection practitioners all outlined concerns that lockdown restrictions may also have made some young people more vulnerable, and in particular increasingly susceptible to grooming and criminal exploitation through County Lines (National Youth Agency 2020; Wedlock and Melina 2020).

In this paper, we seek to inform efforts to safeguard children and vulnerable adults, as the impacts of Covid-19 continue to unfold. Based on interviews with practitioners whose work intersects with County Lines and Child Criminal Exploitation, we assess possible shifts in County Lines offending patterns resulting from measures introduced in response to the pandemic, as well as their impact on efforts to detect, prevent and combat crime, and on the safeguarding of victims of exploitation. The paper attempts to understand how front-line professionals have experienced and perceive the impact of the pandemic on their work and those they engage within the context of County Lines and child criminal exploitation.

### County lines (CL)

The Independent review by Dame Carol Black (Black 2021) estimated that the UK’s illicit drugs market is worth £9.4 billion a year. Substantial national attention has been paid to the evolution of the UK’s drug markets, largely centring on the development and expansion of drug supply networks from urban centres into provincial towns, a process termed ‘County Lines’ (Harding 2020b).

Specifically, the issue of ‘County Lines’ refers to the migration of illegal drugs from urban to rural and coastal areas. Drugs are often transported across different ‘county’ jurisdictions using a branded mobile phone ‘line’, and is often recognised as involving the exploitation of young people and/or vulnerable adults to move and hold drugs and cash (National Crime Agency 2015). The mobile phone is key to CL. Dealing networks typically use mobile phones to establish a database of active drug users, using the phone as a ‘deal line’ which connects new customers to the ‘out of town’ dealers operating in their area (Coomber and Moyle 2018; Harding 2020a, 2020b).

While the term ‘County Lines’ (henceforth CL) has been in use since at least 2015 (National Crime Agency 2015), there remains significant debate (Spicer et al. 2020; Turner et al. 2019) about its inception. Some suggest that the model emerged in response to the saturation of drug availability in major metropolitan areas (Robinson et al. 2019; Windle and Briggs 2015), where a ‘growing number of dealers’ is not matched by growing numbers of dependant users (Ruggiero 2010, 51). Research by Coomber and Moyle (2018) goes further, and suggests that the motivations for ‘outreach supply’ methods like CL are associated with increased opportunities for profit (Van Daele and Vander Beken 2010), and the presence of geographically distant areas where there is a captive market of addicted drug users which are selected to avoid organized competition that might be present in the dealers’ home locales (Windle and Briggs 2015; National Crime Agency 2015, 2016).

Since becoming a national priority in 2018, CL has been officially situated as a form of organised crime. This narrative, reinforced in the Government’s Serious Violence Strategy (HM Government 2018), placed gangs and organised criminal networks at the centre of rising levels of violence, exploitation, and CL activity. In the policing response that followed to reduce CL and its associated harms, territorial police forces have been supported by regional organised crime units (ROCUs) and by the National Crime Agency. In their most recent strategic assessment, the National Crime Agency (2019) associated CL with increases in both gun and knife crime, “an expansion of gangs and organised crime outside of urban centres”, and the increase in the “coercive control and exploitation of vulnerable populations for the purposes of drug dealing” (McLean et al. 2019, 4).

Official definitions of organised crime are vague. The UK’s Serious Crime Act 2015 identifies an organised crime group as that which (1) “has at its purpose, or one of its purposes, the carrying on of criminal activities” and (2) “consists of three of more people who agree to act together to further that purpose”. Yet the extent to which many CL networks are organised is contested. In a ‘rapid appraisal’ of CL in a seaside town in Essex, Jaensch and South (2018: 1) asserted that the gangs involved in CL presented themselves as a “hybrid between traditional street gangs and organised crime groups”, noting that those involved were “visible, known to police and operated at street level where organised criminals tend not to operate” (ibid, 13). While this may hold true for many of the networks involved in CL across the UK, the proliferation of the trafficking of children, the above-mentioned increase in gun and knife crime, as well as rising cases of money laundering and cybercrime (O’Hagan and Long 2019) suggest that CL activity does involve some elements of organised crime.

Indeed, Windle and Briggs (2015a): 1178) observed that drug dealing is separate to gang activity. However, due to the overlap between actors in gangs and CLs it can be hard to establish where a gang ends and where an organised crime group (OCG) begins (Densley, 2012: 44–45). Bonning and Cleaver (2020: 14) assert that gang membership is potentially more likely to be associated with being a perpetrator of violence, while association with county lines is more highly associated with being a victim of it.

The direct attribution of youth violence and drug market participation to gangs has however been contested as problematic (Densley 2011). It is contended that the ‘gang’ narrative continues to be used to legitimise the over-policing of black communities, and contributes to the differential treatment of young black men in the criminal justice system (Williams 2015). Williams also suggests (2015, 19) that the gang label ‘others’ specific groups and communities as problematic and requiring of police intervention. ‘Gang’ rhetoric fails to situate youth violence and CL within broader issues such as socio-economic inequalities, austerity policies and intensified social exclusion (Spicer 2020). The demographics of young people involved in CL suggests that many “come from socially and economically disadvantaged backgrounds” (Windle et al. 2020, 73). Thus, their participation in CL for either money or drugs may actually at times demonstrate a rational form of resilience to these life challenges. That is, young peoples’ involvement in CL can be less an attraction to a criminal lifestyle, but more a representation of their being “instrumental in providing shelter and sustenance for themselves” (Ellis 2018, 161).

### Criminal exploitation in county lines

CL is also increasingly a concern for child welfare agencies, as reports suggest children aged as young as seven are being used as ‘runners’ to move and sell drugs in areas far away from their homes (Turner et al. 2019; Wroe 2019). The use of children in these operations offers distance and anonymity for dealers, who can manage the supply from their local areas without having to remain present in market locations themselves (National Crime Agency 2015). Adults are also known to be exploited as drug ‘runners’ and ‘commuters’, particularly in circumstances where existing drug users are controlled and exploited as ‘user dealers’ (Harding 2020a). There is also acknowledgement of sexual exploitation within some CL environments, particularly where females are involved (Robinson et al. 2019), or indeed through the practice of ‘plugging’ - where drugs are packed and inserted into bodily cavities for transportation (Williams and Finlay 2019; Reginelli et al. 2015).

Criminal exploitation through CL goes beyond the distribution and retrieval of illicit drugs and money, and includes wide range of criminal activities, such as the possession and concealment of weapons, the perpetration of violence (using knives and firearms), the harbouring of offenders, and providing false alibis for others. While these activities vary in terms of level of involvement and severity of offence, the individuals at the root of the criminal exploitation of children and young people are often conceptualised as organised criminals (individuals) and criminal gangs/groups (The Childrens Society 2019). HM Government (2018, 8) contends that child criminal exploitation

“occurs where an individual or group takes advantage of an imbalance of power to coerce, control, manipulate or deceive a child or young person under the age of 18 into any criminal activity (a) in exchange for something the victim needs or wants, and/or (b) for the financial or other advantage of the perpetrator or facilitator and/or (c) through violence or the threat of violence. The victim may have been criminally exploited even if the activity appears consensual.”

Existing research reveals that, despite their exploitation, young people often also exhibit agency and entrepreneurialism in their engagement with CL, seeing it as a way to earn money, gain kudos and maintain their identity with peers (Harding 2020a; Hesketh and Robinson 2019). However, the harms to those involved can be substantial, and they may experience threats to their lives, physical and psychological abuse, and increased involvement with the Criminal Justice System (UK Home Office 2018).

Understandings of child criminal exploitation and CL are still developing. There is a relatively small but growing field of academic literature (Coomber and Moyle 2018; Hesketh and Robinson 2019; Robinson et al. 2019; Spicer 2019; Stone 2018; Windle and Briggs 2015; Windle et al. 2020), in addition to government agency reports, threat assessments and guidance (HM Government 2018; National Crime Agency 2015, 2016, 2017), third sector evaluations (Caluori 2020; Hudek 2018; Rescue and Response 2020; Spicer 2019; Turner et al. 2019; Wroe 2019) and journalistic accounts (Daly 2017; Hymas 2020; Jones 2018) covering the issue. 

The participation of children and young people is often facilitated through the offer of money, luxury consumer items, clothes, drugs and accommodation (Robinson et al. 2019; see also Rees 2011). Debt bondage may be used to maintain control of victims, in which drug debts are used to manipulate and coerce young people to participate in CL operations (Robinson et al. 2019). The centrality of money is also emphasised by Pinkney, who remarks that:

“Young people want things just like we as adults do. But they can’t afford it. They don’t have jobs, there is a lack of resources. CL, robbery, and fraud are quick ways to make money” (Craig Pinkney, quoted in Wedlock and Melina 2020, 53).

Windle, Moyle, and Coomber (2020, 4) offer that CL dealers seek to exploit children because they “represent a cheap, easily recruited workforce”. Young people exploited through CL often present with a number of vulnerabilities, including high rates of missing episodes, being looked after/in the care system, exclusion from mainstream education, and experiences of being victimised and/or having perpetrated serious youth violence - factors which are also associated with increased likelihood of criminal offending (Sturrock and Holmes 2015).

Some young peoples’ appearance as consensual participants in the illegal drug supply contributes to inconsistencies in current criminal justice responses to child criminal exploitation, and official rhetoric about treating children that are identified as involved as victims often does not translate into practice (Wedlock and Melina 2020, 66).

The CL model also often relies on the establishment of a local base, referred to as a ‘trap house’ (Turner et al. 2019). Borrowed from the street term for drug dealing (‘trapping’), these are often residential addresses owned, or rented, by individuals in the drug market area. Properties are subsequently taken over and used to package and distribute drugs – a process referred to as ‘cuckooing’, (Coomber and Moyle 2018; Stone 2018), “named after the nest invading tendencies of cuckoo birds” (Spicer et al. 2020, 2). Cuckooed victims may be specifically targeted, with CL networks seeking out properties belonging to vulnerable adults, such as those with existing addictions, physical disabilities, and severe mental health issues, and the elderly (NCA 2017). Like the criminal exploitation of children, cuckooing has also been framed as a form of modern slavery (Stone 2018).

### County lines during the pandemic

The first national lockdown in March 2020 triggered an immediate drop in reported crime rates across England, with two notable exceptions: drug offences and domestic violence (National Police Chiefs’ Council 2020b). Reductions in violent crime in London, including knife and firearms offences, as well as homicides, were described as a “silver lining” of the pandemic (London Metropolitan Police Commissioner Cressida Dick in Dodd 2020). In the capital, recorded knife crime fell by more than 50% and stab wounds among people under 25 by 69%, causing some to suggest that the pandemic was itself acting as a significant inhibiter of crime (Dodd 2020). A decrease in missing children reports (National Police Chiefs’ Council 2020b) were cited as possible evidence that exploitation linked to CL may have reduced during the lockdown (Caluori 2020). Others argued that the number of missing vulnerable children had in fact *increased* as safeguarding capacity was cut because of the pandemic (Townsend 2020). However, the overall picture is unclear, and changes in recorded incidence may have been a reflection of the pandemic’s impact on reporting behaviour, rather than an actual change in crime prevalence. Moreover, the increase in recorded drugs offences in England and Wales may be a representation of the re-orientation of policing resources towards such ‘visible’ street crimes, as police benefited from the availability of extra resources becoming available due to the curtailing of other policing activities, such as their monitoring of the night-time economy. (Langton et al. 2021).

There remains an absence of clarity regarding Covid-19 attributable shifts in CL behaviour (and in the relative risk it poses to children and young people), reflecting the lack of evidence about changes to illicit drug markets in general. It is perhaps unsurprising that there are no studies predating covid-19 that attempt to establish how a global pandemic might affect illicit drug markets, and the accounts available upon which the pandemic’s impact can be assessed are mostly speculative predictions such as media reports, anecdotal and individual examples, and opinion pieces by sector commenters. It is therefore, “altogether unclear whether (many of) statements derive from some general observations, theoretical argument, or are simply the author’s opinion” (Giommoni 2020, 1).

Whatever the changes in the levels of CL activity and exploitation, young people continued to be found on the country’s rail networks, far away from their homes, and carrying significant sums of money and/or drugs (Grierson and Walker 2020). Despite a 94% reduction in rail travel generally, British Transport Police claimed they had “not seen a reduction” of CL activity involving “juvenile drug runners” on trains (Grierson and Walker 2020). However, it is unclear how this is related to any changes in the level of CL operations themselves, as although fewer young people might be using the rail networks to move drugs, those that remained were increasingly visible to police. Indeed, the British Transport Police acknowledged that the reduction in train services and the requirement to have an essential purpose for journeys made it “incredibly easy” for them to detect and disrupt activity on the rail network (Grierson and Walker 2020).

The increased visibility of children and young people moving drugs on public transport may also have encouraged distribution networks to change their tactics. Superintendent Andy O’Connor of Merseyside police commented in media reporting that drug distribution networks were avoiding public transport and using cars instead (Pidd 2020). This was corroborated by Tidy (2020) who conducted media interviews with middle-tier drug dealers. Moreover, Harding (2020b) detailed how his drug-market involved sources had advised that the supply of drugs from metropolitan centres to provincial or seaside towns now more commonly involved “medium bulk deliveries in cars rather than sending boys down on empty trains and buses, where they would be more likely to be spotted by authorities”. This suggests that ‘runners’ might have been travelling less often, but with larger quantities of drugs and money (Caluori 2020), and remaining in rural or coastal distribution areas for longer.

There was also speculation that drug distribution networks and some drug users anticipated restrictions and began stockpiling drugs in rural and coastal towns before lockdowns were introduced in the UK (Hamilton 2020; Saggers 2020a, 2020b; Tidy 2020). The possibility that children and young people were involved in fewer journeys and longer stays might also have had implications for the number of children reported missing. A reduction in missing children reports may disguise the possibility that those who were missing could have been forced to stay in county bases for longer periods of time (Tidy 2020). This could also serve as an indicator of continued or enhanced cuckooing risk. Interviews conducted by Tidy (2020) also suggested that some drug runners were frustrated at making fewer journeys, as it reduced opportunities to make money, while those in provincial bases were bored and irritated at the prospect of spending extended periods of time in trap houses.

Reductions in the availability of other types of property for use as dealing bases, such as Airbnb accommodation and hotels, might also further increased cuckooing risk (Saggers 2020a, 2020b). However, this change also comes with a trade-off, as increased footfall at residential properties may rouse suspicion, at a time when people were not supposed to be visiting other households (Harding 2020b). Liminal spaces, such as car parks, industrial warehouses and unused railway land, were also cited as locations where drug sales were increasing (National Youth Agency 2020), as was the technique of ‘stacking’, which involves assembling groups of customers together at the same time and place (Caluori 2020).

There were also been reported shifts towards the local recruitment of young people in distribution areas, rather than relying on the traditional model of exploiting people from urban areas, marking the emergence of a new “local franchised model of CL” (Caluori 2020). Reported widely was the use of key worker disguises by runners (Harding 2020b; Pidd 2020), and daily exercise as an excuse for movement (Saggers 2020a, 2020b). Interestingly, there were also reports CLs were practising social distancing, wearing masks and gloves (Lowe 2020), and even refusing to accept cash (Eastwood et al. 2020; Harding 2020b), indicating some adherence to covid-19 protocols.

### Criminal exploitation during the pandemic

Despite the difficulties in measuring prevalence, government, media and sectoral reporting have all been unified in their articulation that the pandemic’s restrictions heightened some vulnerabilities and risks of children and young people to being groomed into, and criminally exploited, through CL. The early stages of lockdown in March 2020 brought fears that children would be targeted by CL networks, as schools and colleges closed, and youth services became severely disrupted or unable to operate entirely. Commentators suggested that these factors would result in a “heightening of risks for those already exploited”, “increasing risks of exploitation”, and the “disruption of response efforts” (Smith and Cockayne 2020). The closure of schools and colleges, as well as much youth service provision, potentially reduced the time that children and young people could spend in relative safety (Coles 2020; National Youth Agency 2020).

Missing from home and care reports also dropped during lockdown (Caluori 2020), with a study by the University of Liverpool reporting a 35% decrease in reports of missing children (Shalev Green et al. 2020). While this may have been expected, concerns remain around the increasing vulnerabilities of those who do go missing, especially children under the care of the local authority, who comprise a significant proportion of all missing cases.

Pandemic-induced periods of lockdown restrictions removed opportunities for professionals to notice possible risks, gather information, and provide space for disclosures, exacerbating the existing ‘postcode lottery’ of service provision, that already leaves many young people at risk (Wedlock and Melina 2020, 82).

The National Youth Agency reported that lockdown was used as a cover for a recruitment drive by criminal networks (National Youth Agency 2020, 7). Increased time spent on the internet was also seen as a potential risk factor for the grooming of children and young people (Harding 2020b; Saggers 2020a, 2020b), especially those previously unknown to child protection services or law enforcement. More time at home meant that some were at increased risk of exposure to domestic violence (National Police Chiefs’ Council 2020a), an issue previously attributed as a push factor towards CL activity (Wedlock and Melina 2020).

CL networks’ tactics may have augmented the vulnerability of children and young people in a variety of ways. National reporting has claimed that gender biases associating CL with males has served to overlook the experiences of girls and women (National Youth Agency 2020; Wedlock and Melina 2020), and it is suggested that females are increasingly used by CL networks to avoid suspicion from law enforcement (National Youth Agency 2020; see also: Saggers 2020a, 2020b). Indeed, the National Crime Agency (National Crime Agency 2017) noted a significant, but not fully quantified, increase in the number of suspected female victims of sexual exploitation under the age of 18, which according to Windle, Moyle, and Coomber (2020, 5) can be “attributed to the rise of CL activity”. Acknowledging the role of agency in female participation, Harding (2020a) coined the term ‘survival narrative’ to refer to an “unexpected change in circumstance[s] lead[ing] women, and men, to engage in behaviours they may not previously have considered”. Such circumstances include, for example, a sudden and unexpected loss of employment, long-term poverty and the UK’s shift from legacy benefits^1^ to Universal Credit which, according to d'Este and Harvey (2020), 19), will worsen the financial conditions of many recipients, and lead to an increase in financially motivated crime.

It is clear the pandemic has had some impact on methods employed to maintain the UK’s illicit drug supply and the associated exploitation of young people. However, it cannot be concluded whether reported changes are speculative, based on anecdotal examples only occurring in one place, regionally variant, or reflective of wider (non Covid-19 specific) trends to avoid detection by law enforcement. Gaps in quantitative reporting mean it is also difficult to ascertain whether reports, which appear to corroborate one another, are drawing on speculative or anecdotal evidence from the same places. Such reporting may obscure the possibility that frontline professionals actually lack full awareness of what has happened to CL operations during Covid-19. Indeed, reduced face-to-face interactions with young people, significant court backlogs and changes in policing priorities are a likely source of much ambiguity. The assumption that CL operations must have changed because of Covid-19 restrictions, the sense of it being an unusual and extraordinary moment, arguably skews the literature away from considering possible continuities.

## Methods

Our research takes a primarily descriptive approach, to develop understanding of a clear and emergent social issue (Silverman 2015, 113) – that of perceived adaptations to CL drug supply and child criminal exploitation during the pandemic. We used qualitative data from interviews with key frontline and analytical practitioners (N = 46), aiming to elicit their experiences of the pandemic to understand perceptions of possible and sudden shifts in perpetrator behaviours, and the resulting emergence of new safeguarding challenges related to CL and CCE. Participants included a) practitioners involved in frontline (statutory or non-governmental) service provision with young people currently or previously involved in CL, or considered at-risk of becoming involved; b) law enforcement officers with portfolio responsibility for policing CL and illegal drug supply; and c) practitioners from law enforcement, non-governmental or other statutory bodies working in analytical roles with responsibility for CL and illegal drug supply. In total, we interviewed 21 police officers (ten from territorial police forces across England, five from within the Regional Organised Crime Units, four from the British Transport Police, and two from the National Police Chiefs Councils), 10 local authority employees, 13 youth workers from non-governmental organisations and two private sector workers. Participants were purposively sampled, and additional participants were identified via a snowball referral approach. Out of the nine English administrative regions where our participants were based, East Anglia and the North East were the least represented locations within our sample. The East Midlands and Greater London were most represented.

Interviews were semi-structured, with flexibility that enabled the exploration of insights on issues where participants had specific first-hand experience or knowledge. All interviews followed the semi-structured template, with slight variances in the phrasing of questions, depending on the participant’s professional role. Interviews lasted between 30 and 90 min, and were recorded using videoconferencing software (Microsoft Teams or Zoom) for transcription by the researchers. Data was initially grouped by the professional role of the participant (police, NGO, Youth Justice, etc.). Data was also cross read to discover and corroborate latent themes (Ryan and Bernard 2000). In some cases, email correspondence was used to elaborate or further clarify points made during the interviews. All interviews were thematically coded (Creswell 2014) giving the researchers flexibility to “identify, analyse, and report patterns (themes) within data” (Braun and Victoria 2006, 6) without being tied to any “pre-existing theoretical framework[s]” (ibid, 9). All coding and analysis was completed by two researchers. Braun and Clarke’s six-step guide to thematic analysis was adopted, enabling the researchers to familiarise themselves with the data by way of transcription, reading and re-reading transcripts, and making notes about general ideas and patterns. Initial codes were generated and arranged into potential themes. Codes included, for example, ‘reduced police intelligence’, ‘dealers using disguises’ and ‘increased social media use’. Potential themes included those that answered the over-arching research question (i.e. how Covid-19 had impacted upon child criminal exploitation and county lines). For example, those relating to ‘safeguarding’, ‘detection’, ‘missing reports’, ‘social media’, etc. The researchers then revisited the interview data in order to review and refine the potential themes, removing any that did not speak to the aims of the research, and merging themes where possible.

The research was not without limitation. Proximity to the Covid-19 pandemic, and the unprecedented nature of major and sustained interruptions to travel and in-person interaction, meant that peer-reviewed academic literature upon which to ground significant proportions of our findings was largely absent, increasing our reliance on other sources, such as journalistic accounts and expert opinions.

Furthermore, we positioned the issue of CL drug supply, and thus our research, as an exploitation (modern slavery) issue. This was made clear in our recruitment materials and meant that prospective participants (for example police) who did not share this framing may have been less inclined to participate. This potentially skews our sample to participants who are more likely to want to express their own promising/effective practices in providing protection for exploited young people, than those who might have insights into the disruption and prosecution of lower-level (exploited) drug runners.

We were also acutely aware that circulating media reports would possibly influence some participants’ views on what changes were (likely) taking place. Therefore, we tried to ensure that questioning was framed so that participants would relay information about actual cases they were involved in as part of their roles. In recognition of this, we probed participants for further details on specific instances to ensure they were not just repetitions or presumptions based on what was being reported elsewhere and in the media. With those in analytical roles this was more difficult – but questions were framed so that participants were asked to relay information from their experience, and we were careful to ask for acknowledgement that relayed information was in-fact representative of that organisation/individual’s experience, and from within their jurisdiction. In geographic areas where we interviewed multiple organisations – we also attempted to corroborate shifts, perceptions, and assumptions across multiple organisations.

In interpreting the results, reasonable assumption was made as to the likely knowledge of the participant based on their current occupation and professional experience. For example, child protection professionals would likely not hold first-hand information on drug market mechanics. Throughout our reporting, we have made effort to ensure that the language used around particular reports and perceptions is indicative of our confidence levels in the information reported to us by participants.

## Results and discussion

Upon thematic analysis of the data, we identified three main themes relating to the effect of Covid-19 on the CL environment. These were (1) perceived adaptations to the CL distribution methodology, (2) the ability of law enforcement to effectively detect and enforce against CL activity, and (3) the impact of lockdown restrictions on the capacity of frontline professionals to efficiently safeguard children and young people involved in CL drug supply.

There was stark contrast between the interviews we conducted with law enforcement participants and those tasked with safeguarding young people. Owing to a generally quieter working space (e.g. closure of the night-time economy and fewer travellers on the rail network), the police appeared enthusiastic in their perceived ability to adapt to lockdown, sharing the many successes that they had experienced in pursuing CL dealers (Dodd 2021), and claiming that they were delivering a “business as usual” service. Conversely, there was a feeling of frustration and disquiet during interviews with practitioners in roles related to youth work. They described having ongoing concerns regarding deteriorating mental health in children and young people, and recognised an increase in the potential for exploitation and harm – particularly via online social media platforms. Participants also commonly commented on a decline in engagement from children with whom they had previously worked to build trust and rapport. The remaining sections will explore these themes further.

### Adaptations to county lines

Across our sample of police participants, it was consistently stated that there was no ‘one size fits all’ adaptation to CL operations in response to Covid-19. We were able to corroborate certain insights across different areas of the country, indicating that some aspects of CL operations had responded similarly to Covid-19 related disruptions. For example, a number of participants believed that cuckooed properties had become potentially easier to detect, making it difficult for networks to set up a base for any significant period of time, and resulting in an increase in ‘day-tripping’ by drug supply lines, rather than establishing a local supply base. One police officer remarked:

“Another issue that a lot of the lines had was that it was becoming too easy to identify the cuckoo addresses from which they were dealing,” they explained. “…before we would see addresses cuckooed for maybe a week, two weeks, and dealers would set up residence, we saw a shift to a quick cycle. So, they’d set up in an address for maybe one [or] two days, and then move because it was too obvious.”

Consistent with media reports (see for example Pidd 2020) our participants reported that CL actors had attempted to use disguises in order to make their travel seem legitimate. Uniforms that included delivery service drivers, supermarket staff, healthcare workers and builders were commonly mentioned by our participants. One police officer described facemasks as “brilliant for CL” as it provided actors with justification to keep their faces covered, and inhibited police familiar with local areas from recognising known individuals.

Police across different areas of the country reported having encountered the use of novel methods of transport to move drugs. It was believed that as supply tactics have the potential to be highly localised, CLs may have responded differently in response to local enforcement efforts and restrictions, and the availability of diverse forms of transportation, infrastructure and local geography. For example, a police officer in the East of England informed us of intelligence suggesting that some networks had relied upon canal barges and drones. Police in the North West of England reported the belief that some networks were reluctant to travel as restrictions added undue risk, and they were instead requesting drug users and commuters to travel to them to collect drugs. This claim was not corroborated with other regions across England.

Across all groups of those we interviewed, it was believed that CL operations were increasingly looking to the use of private rented vehicles to circumvent enforcement, with ‘clean skins’ (people without existing criminal records) and females directed to rent vehicles in person by perpetrators, and the use of online (vehicle) ‘flexi rentals’, either using stolen or fraudulent identification documents. One participant from a car rental business spoke of a typical exploitation scenario that they encountered working in the car rental industry prior to Covid-19:

“So you'd have a young person or a person that's being coerced that would be there trying to arrange for a hire vehicle. They would have someone either on the phone to them, or behind them, basically telling them what to hire, where to hire it, giving them a (credit) card. And you know, more often than not, I'd have fairly switched-on branch staff that would prevent that rental from going out, or would seek advice. But don't get me wrong, there was equally just as many that went through.”

While it is not possible from our research to ascertain the extent to which shifts away from railways were purely pandemic induced, reporting from practitioners was consistent in that, at street level, those moving drugs and completing transactions had been forced to adapt their tactics in order to remain inconspicuous to authorities. The police officers that we interviewed indicated that reductions in footfall along high streets and in residential areas, particularly during the initial lockdown of March 2020, had led to drug runners following the public’s movements, referencing cases where supermarket and shopping centre car parks had been used to avoid detection. Using the cover of daily exercise and key-worker disguises became common methods for hiding in plain sight. As the country’s railways became quieter, it was believed that greater reliance was placed on using the roads to move drugs around, with drug runners themselves conscious of the need to adhere (in appearance at least) to national restrictions and avoid attracting unwanted attention from law enforcement.

Participants working in strategic areas of national law enforcement suspected that the retail price of Class A drugs would increase due to the closure of importation routes and difficulty in accessing supply. It was also suspected that the successes of Operation Venetic (NCA 2020), the National Crime Agency’s campaign as part of an international police operation into the EncroChat encrypted messaging platform (Europol 2020), would have had a noticeable impact on the availability, and thus price, of heroin and crack cocaine (Daly 2020). However, our participants did not report a noticeable change in retail drug prices, mirroring the inelasticity of street-level prices experienced during the 2011 opioid drought (Ahmad and Richardson 2016). Price fluctuations were felt at wholesale, and one police interviewee estimated that the price of cocaine and heroin had risen by up to £10,000 per kilogram in some regions. Similar price rises were also speculated by Saggers (2020a, 2020b). Again, mirroring the effects of the 2011 drought, our police interviewee suggested that those buying drugs in bulk would circumvent retail unit price increases by increasing the use of adulterants, reducing the purity of the drug received by users. Participants in our research did not indicate that the availability of drugs had been disrupted at street level.

While forces acknowledge the exploitation of children for financial purposes, the broader issue of how transactions are made, the location of profits, where they are held, and by whom, remain unclear. One interviewee told us: “[There are] bits of information around card readers… and suggestions of some moving towards cryptocurrencies” to reduce the risk of Covid-19 infection. These suggestions are partially corroborated by Daly (2020). Gaps in participants’ knowledge related to the larger operations behind CLs may also be connected to a lack of recognition and understanding surrounding the middle tiers of drug supply. However, our research did not produce enough evidence to demonstrate whether the pandemic had an effect on these shifts.

### Detection and enforcement

Police participants generally reported confidence in their ongoing ability to detect and carry out enforcement against CL activity throughout the pandemic, taking advantage of a reallocation of resources and relying on neighbourhood policing, increased community intelligence and stronger partnership work. This confidence was expressed most explicitly in engagement with the British Transport Police (BTP), who followed a disruption strategy that focused on making the use of trains increasingly difficult for CL networks. One officer discussed this issue:

“I police the railway. So the numbers on the railway dramatically dropped. Also because the numbers dropped I think there were less people generally... less people running right at the beginning, I think. In terms of people that did get on the trains, they stand out like a sore thumb, because they are going through, we have had cases where they started dressing up as builders go through. The only problem for them is the team recognised them. As always, if there's a reward for the drug market, which there clearly is, there's still going to be people running.”

Indeed, the belief that the BTP were able to spot unaccompanied young people travelling alone provided enhanced opportunities for officers to question their reasons for travel, and they reported being faced with young people in possession of false documentation, incorrect or invalid tickets, and a lack of credible justification for their journeys. The frequency with which young people were being questioned at the beginning of lockdown is a factor that may be considered contributory to the increased reliance of CL on the roads. However, one officer described increased intelligence gathering opportunities when drugs were trafficked via the roads:

“We have seen the movement away from the trains; when they go off the trains, they go on the road again [and] the tactics that we can deploy significantly increase on the road. Because you have cameras, you have a car, you have a vehicle registration number and you have a driver who has a driving licence.”

On the other hand, Covid-19 restrictions made some areas of policing more challenging. Police respondents reported that their ability to conduct interviews with suspects (and the speed at which they could be carried out) was affected due to a lack of available personnel, in particular with lawyers who were working from home or unable/unwilling to attend police stations while self-isolating. Existing backlogs to court processes have been significantly exacerbated by the pandemic (Desroches 2020; House of Lords 2021), and continue to create uncertainty among frontline service providers, increasing the risk to (and vulnerability of) young people who are left in limbo. Some safeguarding practitioners expressed concerns as their caseloads reduced pending trial dates for young people who would likely receive statutory youth service provision following sentencing.

Our findings raise ongoing concerns about the extent to which young people are identified and appropriately supported as victims of exploitation (HMICFRS 2020). Indeed, whether young people involved in CL are referred to the appropriate support services, particularly by police, is unclear, and knowledge of available referral pathways and support among police we interviewed was inconsistent. This is particularly concerning noting the disruption strategies employed across the country’s rail network. However, the police we interviewed did not necessarily indicate to us a reluctance to support victims of CL, but rather a failure to understand the complexities of child criminal exploitation and the available referral pathways. One officer discussed how their own focus had changed:

“When I joined [the Police], I don't think safeguarding was invented yet. All we would do is focus on locking up bad guys. Now the focus [has] really shifted towards safeguarding and away from arrest figures. Senior leaders are still the same. They still want their arrest figures, which looks good. But the thinking is not about the arrest figures for me anymore. It's about a story behind the arrest, and what we do with it… It all sounds good. But what actually happens and what impacts do we actually want? Which is more important?.”

The ongoing effects of austerity also meant that some forces reported working at full capacity, with smaller numbers of staff and with little time and opportunity to undertake training on such matters. Issues of resource availability were notable among our participants agnostic of their professional background, and was cited as an issue independent of the pandemic. Indeed, the impact of austerity measures on public sector services has been well documented (Gray and Barford 2018; Jones et al. 2016; Millie 2013, 2014).

The positive sentiment of the officers we interviewed may be a reflection of limitations in our sampling method, which meant that police officers who felt confident in the victim-centred nature of their response were more likely to respond to our invitations to interview – noting the project’s focus on understanding the implications on young people and criminal exploitation, beyond solely enforcement. Some officers did acknowledge the challenge of working with young people who presented as hostile and/or non-cooperative to police, but who were also likely victims of exploitation in their own right. One officer remarked:

More often than not, we find that we'll arrest [young] people. They won't tell us anything… they'll ‘no comment’ in interviews, they'll leave it up to us to do the legwork. We have to crack the phones. We have to develop the evidence that we've got. And we go into the phones and if we've got someone on the phone in terms of text message saying, you will do this, you've got a debt and you're gonna pay that back by doing this, that and the other. Then we will use the phone work to get the next person along”.

Another police officer also referenced the use of powers that allowed young people to be arrested for their own protection, in order to remove them from the streets. However, ongoing cases where young people are prosecuted for involvement in CL suggest that the positive views of our police interviewees regarding the victim-centred nature of their work on CL is not necessarily pervasive nationally; a view shared among our NGO participants.

### Vulnerability and safeguarding

Our findings indicated that frontline services had perhaps been most directly impacted by Covid-19, due to the complications of providing support and risk assessment during lockdown. Frontline statutory organisations, such as the Youth Justice Service, reported that their resources were stretched even before the pandemic, and the impediments of lockdown had only exacerbated such concerns. Frontline services, including our police and youth offending participants, reported that online and remote working had also given rise to a number of positive outcomes. These included greater flexibility to engage in multi-agency conferences, stronger communication and more cohesive partnership working.

One police participant operating in London remarked that they had observed higher-level perpetrators becoming more involved in the operational aspects of the drug supply, completing journeys in the absence of being able to reliably access young people, as so many were confined at home with parents. This was not a trend reported elsewhere. Despite the drop in missing reports, there was evidence of children and young people considered lower risk being reported missing. In the absence of hard data, one participant speculated:

“Children in care get reported missing a lot quicker than children who are with their parents. Staffing levels in care homes were lower because staff were off-sick or self-isolating, so there was nobody to go and look for these kids, and so there was a lot of reporting of those incidents. Now those incidents, they’re not missing kids, they’ve just gone out to see their mates. They’ve gone out in breach of the direction to stay at home and are reported as missing.”

A small number of our police participants speculated that parents had been less comfortable in disclosing when their children go missing from home, over fears of possible Covid-19 related sanctions. Social care participants reported that they had encountered increasing cases of missing vulnerable children. In either case, changes in recorded incidence may indicate differences in reporting rather than an actual change. Thus, numbers of missing children, and incidences of violent crime, may not proportionately reflect the total level of CL activity.

While most children and young people remained indoors, the use of social media became one of few modes of entertainment. All of the frontline professionals that we interviewed from non-governmental organisations and youth offending teams reported increasing cases of exposure to online harms and abuse across their caseloads, fuelling ongoing concerns that perpetrators are using platforms such as Snapchat and Instagram as part of their coercive repertoire. The glamorisation of drug-related wealth has been proliferated by increased social media use, aiding perpetrators in their ability to groom and attract varying demographics of young people (National Youth Agency 2021). Such platforms were increasingly referenced by the majority of our participants as important in the early stages of grooming. Yet the details of the methods of contact, and the imagery and content used to lure potential victims, were less understood by our sample.

Of particular concern to practitioners in safeguarding roles were reports of increased online sexual exploitation. One of our participants with oversight of youth violence-related A&E admissions across Birmingham, Nottingham and London noted:

“We’ve continued to see incredibly high levels of suicide attempts. What has increased with that is the reason for those suicide attempts being online exploitation.”

Aside from their role in the recruitment of young people, certain social media platforms have been linked to the facilitation of drug supply itself, with Instagram allowing the dissemination of drug-related imagery in large quantities and Snapchat providing quick-fire advertisements that often disappear within seconds (Velten et al. 2017).

Snapchat was also specifically referenced by almost all of our participants as being increasingly important for the logistical movement of drugs to unknown localities, where perpetrators were reported to infiltrate Snapchat groups in the desired location, relying upon young people to map part of their journey and refer their friends for participation in end-user deals. Though there was clear recognition of growth in social media use, participants were typically unable to elaborate on specifics– indicating that it remains a significant knowledge gap among those working directly with young people.

It was reported that young people that remained in remote (online/phone) contact with care providers throughout the pandemic were generally much less comfortable in making disclosures. It was felt among all of those we interviewed in statutory care-facing roles (i.e., social workers and youth offending practitioners) that the restriction to ‘doorstep meetings’ and a general reduction in face-to-face engagement was having a significant impact on their ability to safeguard. Where once professionals reported being able to identify potential indicators of familial harm during meetings outside of the home, they had become restricted in their ability to offer the usual safe environment that encourages engagement and disclosures from young people. Throughout lockdown, many non-statutory organisations were advised to withdraw completely from face-to-face interaction with young people. All of our participants working within non-governmental organisations reported the frustrating loss of engagement with many hard-to-reach young people with whom they had previously spent considerable time building rapport. As a result, many of these young people were deemed ‘lost’ to the grooming tactics of perpetrators, as one participant alluded:

“We’ve lost young people. They’ve totally disengaged and almost committed to that lifestyle now, because the perpetrators are getting a lot more opportunities than we are.”

Overall, the youth workers that we interviewed expressed dissatisfaction at the constraints the pandemic imposed on their ability to effectively safeguard young people. The restriction to doorstep interactions impeded engagement and further challenged efforts to address sensitive issues such as drug (mis)use. There were also concerns among police participants regarding the reduced level of information that they received about at-risk young people from schools and other agencies, with one police force suggesting they had seen a 30% drop in referrals. This reduction contributed to a lack of intelligence to alert law enforcement as to when a child had gone missing.

It was widely perceived by our law enforcement respondents that criminals adapted their practices in response to police activity and the conditions created by lockdown restrictions, making offending harder to detect. The Children’s Society has highlighted that those falling victim to the enhanced tactics of perpetrators include females and university students (France 2020), the latter of which was echoed by one of our police participants in the South East of England. The increased use of females was seemingly also a national trend raised by our sample, but the certainty with which we can attribute that to Covid-19 remains unclear. Statistically fewer females are subjected to stop and search, and thus their exploitation to carry drugs and weapons offers perpetrators greater protection from law enforcement. It is possible that the involvement of females has been increasing without coming to the attention of the authorities. Indeed, in addition to participating at various levels of the CL hierarchy, the exploitation of females for the sexual gratification of male participants is “a normalised and expected part of trap house life” (Harding 2020a, 2020b, 152).

## Conclusion

Clarifications emerging during the initial phases of our research meant that we were able to corroborate some of the issues highlighted by media reporting and other sources. However, the extent and exact impact of Covid-19 in many areas remains unclear. The core findings from our research identify knowledge gaps, particularly regarding the exploitation of children through social media, statutory data collection (e.g., reporting of missing children), and the availability of provisions to protect and safeguard young people. However, in the absence of comparative data, it is difficult to assess whether some of the trends reported throughout the paper are directly a result of lockdown restrictions, or further evidence of gradual changes for which Covid-19 has offered the impetus for them to be noticed.

It also remains difficult to assess whether some of the changes reported by our research participants, and in the literature, are speculative or locality specific, rather than owing to broader pandemic-induced national trends. Despite our efforts to ensure participants were reporting experientially, the lack of frontline administrative reporting and the prevalence of grey literature, it remains difficult to ascertain whether reports which appear to corroborate one another are drawing on speculative or anecdotal evidence from the same places. What is clear is that the impact of Covid-19 continues to amplify existing issues, and is posing additional challenges for practitioners in their work to support vulnerable people and children.

We can say with confidence that Covid-19 restrictions have had an impact on the ability of organisations to safeguard those exploited for the purposes of CL – beyond initial periods of lockdown restriction. Findings from practitioners working in care settings with young people indicate that restrictions continue to exacerbate feelings of isolation and disenfranchisement for many children and young people. Peer recruitment remains important in the referral of other young people to perpetrators, and the apparent ease at which CL networks can infiltrate friendship groups is an ongoing concern and knowledge gap.

While the interpretation of theory can provide a useful framework for which to understand certain sociological issues, Giommoni (2020) argues that there are no theories that have yet been developed to explain drug trafficking (as one example) during a pandemic and as such, caution must be paid to making ‘common sense’ predictions about the impact of Covid-19. We must also exercise caution in our analysis of practitioners’ responses to certain aspects of CLs, and not just shifts in the phenomenon itself. Previous work has identified that the policing of drug markets is particular susceptible to amplification theory – as police officers pursue drugs policing activities that reflect what they believe to be occurring in their local markets (Bacon 2016). Indeed, recent research by Spicer (2021) suggests that factors such as the prioritisation of police responses and the embedded perceptions of officers may be contributors to perceived increases in the prevalence of cuckooing.

Research by Whitfield et al. (2020) highlights similar difficulties in assessing how the pandemic affected levels of drug use, but suggests that apparent reductions in the uptake of regulated needle and syringe programmes may be a worrying indicator that needle and equipment reuse may have increased, exacerbating health risks to drug users (Whitfield et al. 2020). In contrast, research from Australia, while raising similar concerns about the potential health-risks to drug users, also indicates that the pandemic had afforded some innovations - including improving access to drug treatment and safer drug supply, reducing the risk of overdose and incarceration (Grebely et al. 2020).

The extent to which the trends reported in this paper will be experienced in the longer term are still to be tested. While we have attempted to answer some of the ways in which Covid-19 has impacted upon the UK's illicit CL drug market so far, the reality remains that there is limited knowledge of drug markets and organised crime more broadly, both during and post pandemic. The reason for this is simple; Covid-19 has presented scenarios unlike any other experienced by contemporary society.

Notwithstanding this, and despite the limitations of our sample, and the absence of robust data to underpin aspects our findings, our work indicates that the UK’s illicit drug market continues to have a negative impact on communities around the country. The pandemic has posed significant challenges for police and other practitioners, despite reported successes in some areas. Police have had to adapt to the evolving methods and tactics of CL dealers, while themselves working under the constraints of the pandemic. For care practitioners, the pandemic blunted safeguarding tools, causing significant ongoing concerns about the safety and wellbeing of children and young people. However, it is clear that regardless of the extent to which pandemic conditions have exacerbated the prevalence of certain activities linked to County Lines, their very presence further evidences the need to addresses the systemic issues and inequalities that lay at the root of exploitation.

## Footnotes

1 The six legacy benefits being replaced by Universal Credit include: Housing Benefit, income-related Employment and Support Allowance (ESA), income-based Jobseeker’s Allowance (JSA), Child Tax Credits (CTC), Working Tax Credits (WTC) and Income Support
